# Galectins in Cancer and Translational Medicine: From Bench to Bedside

**DOI:** 10.3390/ijms19102934

**Published:** 2018-09-27

**Authors:** Armando Bartolazzi

**Affiliations:** 1 Pathology Research Laboratory, St. Andrea University Hospital, via di Grottarossa 1035, 00189 Rome, Italy; armando.bartolazzi@ki.se or a.bartolazzi@sanita.it; Tel.: +39-065-994-5778; Fax: +39-065-994-5331; 2 Ministry of Health—Lungotevere Ripa 1, 00168 Rome, Italy

Galectins (also worded S-type lectins) are an evolutionarily conserved family of carbohydrate-binding proteins characterized by the presence of β-galactoside-binding sites, generally represented by one or two 130 amino acid carbohydrate recognition domain (CRD). No other types of folded protein domains are structurally present [1,2]. These molecules are functionally involved in several physiological processes, among which inflammation, immune-response, RNA splicing, gene transcription, apoptosis, signaling, cell migration, and differentiation [3]. Moreover, these pleiotropic molecules are also in the position to play relevant biological roles in different diseases, among which fibrosis, cancer, and heart diseases deserve a special mention [3]. Galectins can be found both intracellularly and in the extracellular milieu and are functionally active in converting glycan-related information into cell biological programs. Many biological activities of galectins, in fact, are mediated by carbohydrate-dependent interactions with glycoconjugates, mostly occurring extracellularly. Galectins-mediated extracellular functions require the CRD binding to saccharides associated with cell surface glycoproteins, for example, on T-lymphocytes, stromal cells, and endothelial cells (i.e., TCR, integrins, CD44, CD36, CD13, gangloside GM1) and/or to extracellular glycoproteins (i.e., laminin, fibronectin, vitronectin, elastin, tenascin, lymphokines). Considering the fact that different cell types (both normal and malignant cell types) generally express a specific “galectin signature”, it appears that the overall function of different galectins can vary considerably according to the biological context. Specific galectins in fact can modulate the T-cell immune-response, and galectin-1 has been reported to induce T-cell apoptosis via interaction with TCR, whereas galectin-3 suppresses apoptosis and increases T-cell proliferations. The interaction of galectins with specific extracellular matrix components (glycoproteins) may affect, in turn, cell adhesion, migration, and homing. This is particularly important for tumor cells, which use specific galectin-ligand interactions for promoting tumor growth, progression, and immune-escape [2,3,4].

However, several intracellular functions mediated by galectins seem to be carbohydrate-independent (i.e., interactions with cytosolic or nuclear targets). This has been demonstrated in particular for galectin-3, which was found in the nucleus and cytoplasm of different cell-types. Galectin-3 interacts with Ras and Bcl-2 in the cytosol and these molecular interactions play a key role in regulating cell growth and apoptosis. Interestingly, such galectin-3-mediated functions can be abrogated by carbohydrate interference. This finding opens an interesting scenario on the possibility to target specific galectins for therapeutic purposes [4,5].

In cancer, altered glycosylation is a common finding, both on the tumor cell surface and in the extracellular matrix, and the discovery of “tumor specific galectin signatures” opens a fashionable scenario. Many molecular interactions mediated by galectins, in fact, could be functionally relevant for tumor growth and progression. It is not surprising indeed that scientists are going to look “in the sugar box” in order to identify potential diagnostic and predictive tumor markers. At the same time, researchers are dissecting, at the structural and functional levels, those functionally relevant galectin-mediated molecular interactions that are critical for tumors and can be potentially targetable with tailored neo-glycoproteins, glycomimetics, or other biological tools. It is likely that new avenues for cancer therapy will be explored in the near future [3,4,5,6,7].

As aforementioned, cancer does not represent the only playground in which galectins works. Several physiological processes seem to be regulated by galectins. Regulation of immune response, bone cell differentiation, and macrophages activation just represent some of the examples. A large number of papers recently published in the literature also support the notion that the biological relevance of galectins is not restricted to cancer. Galectins have been discovered to be functionally active in many different diseases, some of which deserve special attention for the possibility to target these molecules for a therapeutic intervention [8,9,10].

In this special issue, focused on galectins in translational medicine, a total of 20 interesting papers, 15 of which are represented by comprehensive reviews and 5 original research studies, have been finally considered. These contributions are clustered into 4 groups: (1) Structural and functional studies on galectins and ligands; (2) Galectins and cancer; (3) Galectins in non- neoplastic diseases; and (4) Miscellanea, as detailed in Table 1.

The first two papers [11,12] belong to the structural cluster. Research on galectin glycobiology has drawn much attention for the multifunctional features of these molecules, which can be considered potential targets for selective therapeutic interventions in many pathological contexts. The elegant report by Chan at al. [11] provides an in-depth review on galectin inhibitors, focusing on the structure-activity relationship with the attempt to clarify how these potential therapeutic tools interact with galectins. Structural and functional studies on the galectin-ligand interaction represent an important and necessary step for developing new tools tailored to bind galectins and to eventually block galectin-mediate functions. Bumba et al. [12] report a molecular model focused on the galectin-3 carbohydrate recognition domain. This specific study analyzes the mode and kinetics of galectin-3 bindings to a panel of multivalent neo-glycoproteins, which present complex poly-LacNAc-based oligosaccharide ligands on albumin scaffold. Significant differences in the binding kinetics are observed among the different glycoproteins. A tetrasaccharide capped with *N*,*N*′-diacetillactosamine (LacdiNAc) showed the strongest ligand ability to galectin-3. This information may drive the development of neo-glycoproteins ingegnerized ad hoc to specifically interfere with galectin-3 mediated functions.

The cluster worded “Galectins and Cancer”, as expected, is the larger one. The cluster includes several papers focused on cancer diagnosis, prognosis, and galectin-based therapeutic approaches [4,5,6,7,13,14,15,16,17,18,19,20,21]. The implication of galectins in tumor growth and progression, and more in general the impact that these molecules may have in oncology, is very well-known and has been extensively reviewed [3]. More specifically, for this paper collection, Li et al. [13] demonstrate the possibility to identify deregulated proteins, functionally related to galectin-1, by using an in vitro model of urinary bladder urothelial carcinoma and a proteomic approach. Modulation of these galectin-1 related molecules may have prognostic value for this tumor type. Similarly, a specific galectin signature, which can be used as prognostic marker, has been also identified in ovarian carcinoma [14,15].

Thyroid cancer represents the paradigmatic tumor model, in which experimental studies on galectins glycobiology, in particular on galectin-3, contributed greatly to improving cancer diagnosis. A validated galectin-3 test-method for the preoperative detection of thyroid cancer has been already translated in the clinical setting and is changing the clinical management of patients with thyroid nodules, reducing consistently unnecessary surgical procedures [24,25,26,27,28,29]. Three contributions in this specific field, showing different clinical experiences, are presented here [16,17,18]. A novel galectin-3-based immune-positron emission tomography (immune-PET) for imaging thyroid cacer in vivo, and its biological rationale is also presented and discussed [18,30,31]. One of the main functions of galectins in cancer is the regulation of tumor cell adhesion and migration. Modulation of these functions is likely relevant for tumor growth and progression. Galectin-7 is abnormally expressed in epithelial tumors and is involved in tumor progression and metastasis. Advedissian et al. [19] investigated the physiological function of this lectin in epithelial cells and demonstrated its aberrant expression in malignant epithelial counterparts. Studies on the functional role of galectins in neuroblastoma [7] and multiple myeloma [20], two relatively rare tumor conditions, are also presented in this special issue. Up-regulation of galectin-1 has been previously reported in aggressive neuroblastoma, the most common extracranial tumor of childhood. Galectin-1 is necessary for balancing immune response and angiogenic processes in physiological conditions, but cancer exploits these functions to escape from attacks of the host immune system and to better survive in hypoxic conditions. A strategic approach that combines radiotherapy and galectin-1 blockade is proposed and may be useful for reactivating an efficient tumor immune response [7]. Multiple myeloma are characterized by the tight adhesion between neoplastic plasma cells and a bone marrow microenvironment that allows for tumor cell survival and drug resistance. In this niche, neo-angiogenesis is induced as well as immunosuppression and osteolytic metastasis. In the review by Storti et al. [20], the authors analyze the expression profile of different galectin molecules and their functional activities in these specific tumors, with special focus on tumor progression, angiogenesis, and osteoclastogenesis. Several of the identified galectins may be attractive targets for multiple myeloma therapy. The expression of galectin-1 and -8 on tumor cells in fact, correlate with patients’ survival.

Finally, three very interesting and comprehensive reviews exploring new potential therapeutic strategies for cancer treatment, based on galectin targeting, are considered in this book [4,5,6]. This paper represent a hot topic in oncology. Structural and functional studies on galectin-ligand interactions performed at molecular level, in fact, may produce important information for creating therapeutic interventions based on galectin-targeting. The proposed therapeutic approaches are supported by a strong biological rationale. New therapeutic strategies for cancer may involve the use of specific galectin inhibitors, such as competitive carbohydrates, small glycomimetic molecules, and monoclonal antibodies to specific galectins, that can be used alone or in combination with other therapeutic options.

An interesting issue is also represented by the fact that tumor-derived galectins may have bifunctional effects on tumor cells and immune cells. This has been extensively studied for galectin-1, galectin-3, and galectin-9 in different cancer types. In the review by Chou et al. [6], this issue is specifically discussed.

A short communication by Koonce et al. [21] reports the biological effects of a non-peptidic galectin-1 allosteric inhibitor, worded OTX008 and able to induce tumor vessel normalization and tumor growth inhibition in squamous cell carcinoma models. These effects are related to the improved tumor oxygenation registered after treatment with such a galectin-1 inhibitor.

This condition allows a more permissive tumor microenvironment status for improved radiation or chemotherapeutic treatment.

The cluster entitled “Galectins in Non-Neoplastic Diseases” includes three interesting reviews. The first by Sciacchitano and co-authors [22] is focused on galectin-3. The authors report in an elegant and original way a large number of studies published so far on the role of galectin-3 in different diseases, listed in alphabetical order. This original contribution provides the base for further investigations in each specific medical field.

It has been previously reported that galectin-3 is required for TGF-β mediated myofibroblast activation and matrix production. Increased galectin-3 expression seems to be a common feature in tissue fibrosis. Disruption of the galectin-3 gene blocks myofibroblast activation and procollagen I expression, in an experimental model of liver fibrosis in vitro and in vivo [23].

Clementy et al. described the role of galectin-3 in the pathogenesis of atrial fibrillation [8]. Considering the fact that galectin-3 is a well-recognized biomarker of fibrosis and tissue remodeling, the authors discuss the mechanisms of such an electrical alteration and its therapeutic implications.

A very interesting review published by Lai and co-authors [9] is focused on the translational implication of galectin-9 in the pathogenesis and treatment of viral infection. The interaction between galectin-9 and the ligand Tim-3 triggers signaling events that regulate immune response. Galectin-9 has been reported to be over-expressed in a variety of target cells infected by different viruses, among which include HCV, HBV, HSV, influenza virus, dengue virus, and HIV. The biological significance of galectin-9 expression in virus infected cells and its functional implications in regulating virus specific immune-response also represent important fields of research to be pursued. Understanding the biological role of galectin-9 in this specific context may help to identify better methods for monitoring and treating viral infections. The last cluster of contributions includes the work by Iacobini et al. [10], where they reported original data on galectin-3’s biological role in all stages of bone biology, from development to remodeling. The regulatory role of galectin-3 in inflammatory bone and joint disorders is also discussed, together with the possibility to target galectin-3 for therapeutic purposes.

The special review issue on the recently discovered galectin-12 and its functional role in regulating important physiological mechanisms, like cellular differentiation, apoptosis, and macrophage polarization to M2 subtype [32], closes on galectins and translational medicine. Although galectin biology is attracting the interest of many scientists operating in different experimental contexts and clinical fields, from developmental biology to clinical oncology, many aspects concerning galectins are still incompletely understood and need further investigation. Among these, some deserve mention: elucidation of galectin-mediated intracellular mechanisms; galectin-dependent signaling; structural aspects of galectin functions (i.e., oligomerization, galectin-derived extracellular lattice); and the biological and functional significance of specific “galectin signatures” in benign and malignant tissue as well. Addressing these issues will open the way to new therapeutic strategies for different human diseases, stimulating research for developing glycomimetics, neo-glycoproteins, and/or monoclonal antibodies directed to specifically modulate or block the functions of these fascinating molecules.

Overall 20 important contributions published in this special issue illustrate recent advances in the field of Galectins, which may have potential clinical implications in cancer but also in other clinical contexts. It seems to me that an interesting chapter of translational glycobiology is on the horizon.

I would like to thank all the authors who contributed to this special issue and I remain hopeful that the increasing knowledge in the field will require, in the very near future, a new and more extensive effort for preparing the second volume entitled “Galectin in cancer and translational medicine II”.

## Figures and Tables

**Table 1:** Contributions to the special issue “Galectins in Cancer and Translational Medicine: From Bench to Bedside”.

Cluster	Paper	Reference
**(1) Structural and Functional Studies on Galectins and Ligands**	Dissecting the structure-activity relationship of galectin-ligand interactions	Chan Y.-C. et al. [11]
	Poly-N-acetyllactosamine neo-glycoproteins as nanomolar ligands of human galectin-3: binding kinetics and modeling	Bumba et al. [12]
**(2) Galectins and Cancer**	Proteomic identification of the galectin-1-involved molecular pathways in urinary bladder urothelial carcinoma	Li C.F. et al. [13]
	Overall survival of ovarian cancer patients is determined by expression of galectins-8 and -9	Schulz H. et al. [14]
	Galectin-1, -3, and -7 are prognostic markers for survival of ovarian cancer patients	Schulz H. et al. [15]
	Galectin-3 performance in histologic and cytologic assessment of thyroid nodules: a systematic review and meta-analysis	Trimboli P. et al. [16]
	Galectins and carcinogenesis: their role in head and neck carcinoma and thyroid carcinomas	Kindt N. et al. [17]
	Galectin-3: the impact on the clinical management of patients with thyroid nodules and future perspectives	Bartolazzi A. et al. [18]
	Galectin-7 in epithelial homeostasis and carcinoma	Advedissian T. et al. [19]
	TrkB-target galectin-1 impairs immune activation and radiation responses in neuroblastoma: implication for tumor therapy	Batzke K. et al. [7]
	Role of galectins in multiple myeloma	Storti P. et al. [20]
	Galectin targeted therapy in Oncology: Current knowledge and perspectives	Wdowiak K. et al. [5]
	Role of galectins in tumors and in clinical immunotherapy	Chou F.-C. et al. [6]
	Galectins as molecular targets for therapeutic intervention	Dings R.P.M. et al. [4]
	Galectin-1 inhibitor OTX008 induces tumor vessel normalization and tumor growth inhibition in human head and neck squamous cell carcinoma models	Koonce N.A. et al. [21]
**(3) Galectins in Non-Neoplastic Diseases**	Galectin-3: one molecule for an alphabet of diseases, from A to Z	Sciacchitano S. et al. [22]
	Galectin-3 in atrial fibrillation: mechanisms and therapeutic implications	Clementy N. et al. [8]
	Translational implication of galectin-9 in the pathogenesis and treatment of viral infection	Lai J-H. et al. [9]
**(4) Miscellanea**	Role of galectin-3 in bone cell differentiation, bone pathophysiology and vascular osteogenesis	Iacobini C. et al. [10]
	Galectin-12 in cellular differentiation, apoptosis and polarization	Wan L. et al. [23]

## Abbreviation

CRD	Carbohydrate recognition domain
LacdiNAc	*N*,*N*′-diacetillactosamine
HCV	Hepatitis C virus
HBV	Hepatitis B virus
HSV	Herpes simplex virus
HIV	Human immunodeficiency virus
TGF-β	Transforming growth factor-β
Tim-3	T-cell immunoglobulin domain, mucin domain-3
